# The effects of sex hormones during the menstrual cycle on knee kinematics

**DOI:** 10.3389/fbioe.2023.1209652

**Published:** 2023-09-05

**Authors:** Zhou Bingzheng, Zhao Xinzhuo, Jin Zhuo, Yang Xing, Li Bin, Bai Lunhao

**Affiliations:** ^1^ Department of Orthopaedic Surgery and Sports Medicine, Shengjing Hospital of China Medical University, Shenyang, China; ^2^ Department of Biomedical Engineering, Shenyang University of Technology, Shenyang, Liaoning, China; ^3^ Center of Reproductive Medicine, Department of Obstetrics and Gynecology, Shengjing Hospital of China Medical University, Shenyang, China; ^4^ Research Center for Universal Health, School of Public Health, China Medical University, Shenyang, China

**Keywords:** menstrual cycle, sex hormones, kinematics, estradiol, progesterone, anterior cruciate ligament, non-contact injury

## Abstract

The effects of the menstrual cycle and sex hormones on knee kinematics remain unclear. The purpose of the study was to investigate the effects of the menstrual cycle and serum sex hormone concentrations on knee kinematic parameters of the 90°cutting in female college soccer athletes. Three female college soccer teams (53 subjects) participated in the study. During the first menstrual cycle, a three-step method was used to exclude subjects with anovulatory and luteal phase–deficient (LPD) (12 subjects). The subjects’ menstrual cycle was divided into the menstrual phase, late-follicular phase, ovulatory phase, and mid-luteal phase (group 1, 2, 3, 4). In each phase of the second menstrual cycle, we used a portable motion analysis system to enter the teams and tested the sex hormones concentrations and knee kinematics parameters in three universities in turn. We found that subjects had a lower maximum knee valgus in group 4 compared with other groups. This meant that subjects had a lower biomechanical risk of non-contact anterior cruciate ligament (ACL) injury in the mid-luteal phase. There was no significant correlation between serum estrogen, progesterone concentration, and knee kinematic parameters. This meant that sex hormones did not have a protective effect. Future studies need to incorporate more factors (such as neuromuscular control, etc.) to investigate.

## 1 Introduction

ACL injury is one of the most common sports injuries, and the incidence of female athletes is higher than that of male athletes (Voskanian, 2013). Most ACL injuries are non-contact injuries (Renstrom et al., 2008; Alentorn-Geli et al., 2009). Studies on the classification of sports have found that changing direction actions or jumping is considered a high-risk action for non-contact ACL injury in high-risk sports such as soccer, basketball, rugby, handball, and volleyball (Montalvo et al., 2019). Biomechanical studies on the changing direction actions have also found that the knee would produce multi-plane kinematics load, which would affect the stress of ACL (Fox, 2018).

Although the effect of the menstrual cycle on knee kinematics has been studied for decades, the conclusions are still not unified. The contradiction is mainly reflected in whether kinematics parameters are different in different phases. At present, the corresponding investigation mainly focuses on testing high-risk actions for non-contact ACL injury (e.g., cutting, landing, jumping, and so on) (Chaudhari et al., 2007; Dedrick et al., 2008; Park et al., 2009a; Park et al., 2009b; Shultz et al., 2011; Shultz et al., 2012; Okazaki et al., 2017; Gilmer et al., 2020; Gilmer and Oliver, 2020).

In studies of landing actions, the main contradiction was whether kinematics parameters were different before and after ovulation (Chaudhari et al., 2007; Dedrick et al., 2008; Cesar et al., 2010; Shultz et al., 2011). Studies with significant differences indicated that subjects appeared to have a higher biomechanical risk of non-contact ACL injury before ovulation (Cesar et al., 2010; Shultz et al., 2011). However, only one of the above studies excluded anovulatory and LPD (anovulatory: 2/26; LPD: 4/26) (Dedrick et al., 2008). In studies of jumping, cutting, stopping, and other actions, the conclusions were that knee kinematics parameters were not different in different phases. However, the above studies did not exclude anovulatory and LPD (Park et al., 2009a; Park et al., 2009b; Shultz et al., 2012; Okazaki et al., 2017; Gilmer et al., 2020; Gilmer et al., 2020). Therefore, using a standardized menstrual cycle methodology is a prerequisite for conducting such a study in the future (Hewett et al., 2007; Balachandar et al., 2017; Somerson et al., 2019).

Several reviews (Janse et al., 2019; Elliott-Sale et al., 2021; Schmalenberger et al., 2021) have suggested that although serial follicular ultrasound scanning is the gold standard for excluding anovulatory, the indirect method of combining menstrual calendar mapping with urinary pregnancy tests can effectively exclude anovulatory. Whereas for LPD, the invasive method is still needed. Schaumberg et al. (2017) recommended a three-step method to exclude anovulatory and LPD among the subjects and provided validity validation. In addition, some scholars suggested that more attention should be paid to exploring the effect of serum concentrations of sex hormones rather than just focusing on the different phases of the menstrual cycle (Burden et al., 2021).

To reduce the frequency of blood collecting and experimental costs, the study was conducted in two menstrual cycles. In the first menstrual cycle, the “three-step method” was used to exclude anovulatory and LPD, and the subjects’ menstrual cycle was divided into the menstrual phase, late-follicular phase, ovulatory phase, and mid-luteal phase. In the second menstrual cycle, serum sex hormone concentrations and knee kinematic parameters were tested synchronously. We hypothesized that knee kinematics parameters would be different in different phases when subjects performed 90° cutting, and serum sex hormone concentrations were correlated with knee kinematic parameters.

## 2 Materials and methods

### 2.1 Subjects

We recruited female college soccer athletes from three universities in a city in northern China. Subjects were required to have a BMI from 18.5 to 23.9 and had no history of sports injury or pregnancy. Subjects had no history of contraceptive use and self-reported consistent menstrual cycles (25–38 days) within the 6 months before the study. Finally, fifty-three female college soccer athletes who met the criteria voluntarily participated in the study. All subjects received written and verbal information about the study and gave written informed consent before the study. The study was approved by the institutional review board and conducted according to the Declaration of Helsinki.

### 2.2 Division of the menstrual cycle

Subjects completed the study within two menstrual cycles. In the first menstrual cycle, the onset day of the menstrual phase, ovulatory day, and cycle days were recorded according to the bleeding and ovulation prediction kit (Diagnostic Kit for luteinizing Hormone Colloidal Gold, ACON Biotech, Hangzhou, Inc., China). The onset day was defined as the first day of bleeding and the ovulatory day was defined as 2 days following the ovulation prediction kit was positive. On the day after the bleeding stopped, morning urine samples were tested continuously daily until the ovulation prediction kit was positive. Subjects took photos of the test using their smartphones and sent the picture to the study manager, who verified whether the test was positive or not. Subjects whose ovulation prediction kit was not positive were regarded as anovulatory and were withdrawn from the study.

The mid-luteal day was defined as the median number of days during the luteal phase. At 6:30 on the mid-luteal day, researchers went to school to test subjects’ serum progesterone concentrations. 8 mL of the blood samples were collected from the antecubital area of the subjects’ arms on an empty stomach. The blood samples were transferred to a 10 mL polypropylene tube and then sent to the laboratory. The blood samples were centrifuged at 1,500 g for 15 min, and the serum was separated for detection. The detection instrument was the automatic particle chemiluminescence immune analyzer (UnicelTM DXI800Accsss, Beckman Coulter, Inc., United States), and the detection kit was purchased from the company. The detection process strictly followed the instructions. LPD was defined as a progesterone concentration <6 ng/mL and was withdrawn from the study.

In the second menstrual cycle, subjects were scheduled for the study on specific days.

(1) Day 2 of the onset day of the menstrual cycle;
(2) Days 2 before the ovulatory day;
(3) The ovulatory day;
(4) The median day of the luteal phase.

The specific days were defined as the specific phases. We defined the menstrual phase as group 1, the late-follicular phase as group 2, the ovulatory phase as group 3, and the mid-luteal phase as group 4.

### 2.3 Test of sex hormones and kinematics

**FIGURE 1:**
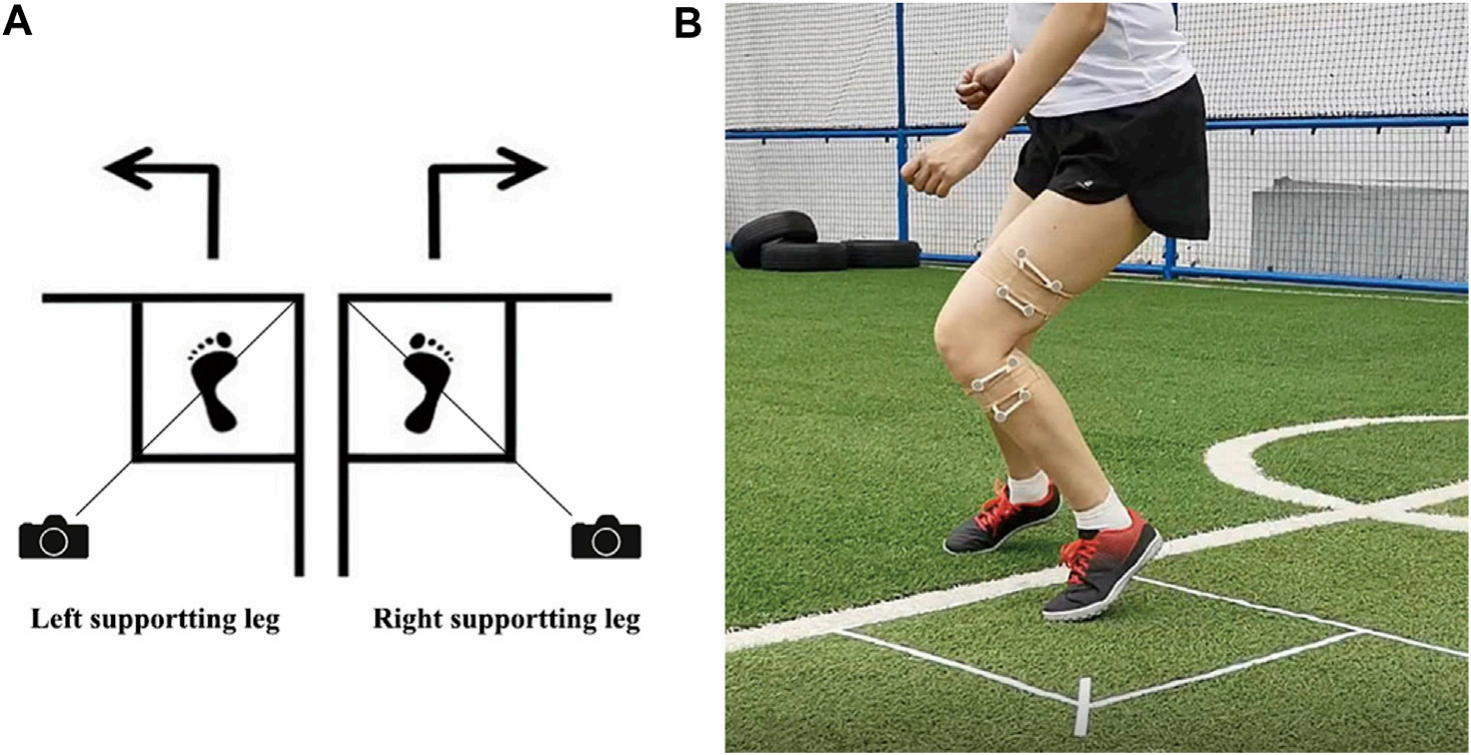
**(A)** Schematic diagram of 90° cutting of subjects. Draw a square foot box, take the diagonal of the square, and set the camera on the diagonal. Subjects select the supporting leg based on their own habits; **(B)** Mark position of infrared light-reflecting markers.

For the test of the second menstrual cycle, we conducted the test at three universities in sequence. On the specific days of the second menstrual cycle, serum sex hormone concentrations and knee kinematic parameters were tested synchronously. The steps of the serum sex hormone concentrations test were the same as before, and kinematic parameters were tested at the subjects’ training turf at 8:00. Before the test, subjects took a regular warm-up exercise. Subjects chose the supporting leg to perform 90° cutting and were allowed several preparation trials (Figure 1A).

A portable marker-based motion analysis system (Opti_Knee^®^, innomotion inc., Shanghai, China) was used to record the angular displacements of each plane. An integrated synchronous high-speed camera (Basler aca640-90uc, Basler AG) was used to capture video activity. A workstation computer with customized software (Opti-Knee V1.0, Shanghai Innomotion inc.) was used to perform real-time calculations parallelly. Subjects’ thighs and shanks were attached with two rigid plates, each composed of four infrared light-reflecting markers (OK_Marquer1, Innomotion Inc., Shanghai, China) (Figure 1B).

Subjects’ greater trochanter, lateral epicondyle, medial epicondyle, lateral plateau, medial plateau, tibial tuberosity, fibular head, medial malleolus, and lateral malleolus were identified by a handheld digitizing probe. Subjects were positioned within the capture area of the system, with all reflective markers being clearly detected by the system when subjects performed 90° cutting. Data were collected for each frame using the geometric relationships between the reflective markers under the femur and tibia coordinate systems that were established during calibration. Anatomic frames on the femur and tibia were defined based on the respective bone landmarks. The femoral origin was located at the midpoint of the transepicondylar axis, a line connecting the prominent points of the medial and lateral femoral epicondyles. The medial-lateral axis followed the transepicondylar line. The anterior-posterior axis was perpendicular to the plane defined by the transepicondylar line, and the greater trochanter, and the proximal-distal axis was set perpendicular to the other two axes. The midpoint of the line connecting the most medial and lateral points of the tibial plateau was defined as the origin of the tibial coordinate system. The medial-lateral axis followed the medial and lateral tibial plateau line, the anterior-posterior axis was perpendicular to the plane defined by the medial-lateral axis and the lateral malleolus, and the proximal-distal axis was set perpendicular to the other two axes. The three-dimensional (3D) trajectory of the rigid bodies during activities was tracked by the stereo-infrared camera at a frequency of 60 Hz with an accuracy of 0.3 mm root mean square. The 3D positions of the identified femoral and tibial anatomical landmarks were calculated based on their geometric relationship to the femoral or tibial rigid plates, respectively. Local femur and tibia coordinate systems were then established based on these landmarks. The 3D translation was quantified as the relative displacement between the origins of the femur in the tibial coordinate system. Similarly, the angular rotations were determined by the femoral coordinate system with respect to the tibial coordinate system. The portable motion analysis system has a repeatability of less than 0.9 mm in translation and 1.3 mm in rotation.

Video activities were captured by an integrated synchronous high-speed camera, and real-time calculations were performed by a workstation computer with customized software parallelly. The landing time and leaving time were identified by manually recognized captured video. The data were extracted between the landing time and leaving time. The data during actions were normalized, and the peak values were identified.

### 2.4 Statistical analysis

The Kolmogorov-Smirnov test was conducted to examine the distribution of serum sex hormone concentrations and knee kinematic parameters. One-way repeated measures analysis of variance (ANOVA) was used to examine mean serum sex hormone concentrations and knee kinematic parameters in four groups. LSD pairwise comparisons test for multiple comparisons was used to compare means of variables between any two different groups, and the level of statistical significance was set at *p* < 0.05. Simple correlation analysis was used to determine the relationship between serum sex hormone concentrations and knee kinematic parameters in four groups, and the level of statistical significance was set at *p* < 0.05.

## 3 Results

### 3.1 Anovulatory and luteal phase-deficient

Seven (13.0%) subjects were anovulatory, fifteen (27.8%) subjects were LPD, and thirty-one (57.4%) subjects finally completed the study (Table 1). The distribution of serum sex hormone concentrations and knee kinematic parameters was approximately normal in the four groups (*p* > 0.05). The Mean and Standard Deviation (SD) of the serum sex hormone concentrations and knee kinematic parameters in four different groups were listed in Table 2.

**TABLE 1:** Basic information of the subjects (Mean ± SD).

Subjects (53)	AGE Y)	HEIGHT (cm)	MASS (kg)	BMI
LPD 7)	18.1 ± 0.6	171.1 ± 3.2	56.6 ± 3.0	19.3 ± 0.6
Anovulatory (15)	18.4 ± 0.6	170.7 ± 3.6	57.2 ± 3.0	19.6 ± 0.7
Normal (31)	19.6 ± 1.3	171.7 ± 6.4	64.6 ± 5.0	21.9 ± 0.6

**TABLE 2:** Serum sex hormone concentrations and knee kinematic parameters in different groups during the menstrual cycle (Mean ± SD).

	Group 1	Group 2	Group 3	Group 4	F	*p*
Estrogen (pg/mL)	53.0 ± 8.4 ^bcd^	160.9 ± 34.8 ^acd^	56.9 ± 5.6 ^abc^	118.7 ± 26.1 ^bcd^	381.0	0.000^a^
Progesterone (ng/mL)	0.8 ± 0.3 ^bcd^	0.9 ± 0.2 ^acd^	0.9 ± 0.3 ^abc^	14.0 ± 2.2 ^bcd^	1,313.0	0.000^a^
Max flexion (deg)	63.1 ± 5.2	61.5 ± 7.0	61.9 ± 7.1	62.1 ± 7.6	1.304	0.279
Max extension (deg)	17.1 ± 2.7	17.1 ± 2.8	17.1 ± 2.6	16.8 ± 2.6	2.855	0.055
Max varus (deg)	−1.3 ± 1.7	−1.3 ± 1.7	−1.3 ± 1.7	−1.3 ± 1.6	0.322	0.760
Max valgus (deg)	6.6 ± 3.0 ^days^	6.7 ± 2.8 ^days^	6.7 ± 2.9 ^days^	6.0 ± 2.8 ^abc^	72.540	0.000^a^

^a^ Statistically significant difference compared with other groups (*p* < 0.05). a Statistically significant difference compared with group 1 (*p* < 0.05), b Statistically significant difference compared with group 2 (*p* < 0.05), c Statistically significant difference compared with group 3 (*p* < 0.05), d Statistically significant difference compared with group 4 (*p* < 0.05).

### 3.2 Serum sex hormone concentrations

Serum estrogen concentration was significantly different (*p* < 0.05). The lowest concentration was observed in group 1 (53.0 ± 8.4 pg/mL), and the first and second peak of concentration was observed in group 2 and group 4 (160.9 ± 34.8; 118.7 ± 26.1 pg/mL). Serum progesterone concentration was significantly different (*p* < 0.05). The lowest concentration was observed in group 1 (0.8 ± 0.3 ng/mL), and the peak concentration was observed in group 4 (14.0 ± 2.2 ng/mL) (Table 2).

### 3.3 Knee kinematic parameters

In the sagittal plane, the maximum flexion and extension angle were not significantly different (*p* > 0.05) (Table 1; Figure 1). In the frontal plane, the maximum varus angle was not significantly different (*p* > 0.05), while the maximum valgus angle was significantly different (*p* > 0.05). The lowest maximum valgus angle was observed in group 4 (4.9° ± 4.0°) (*p* < 0.05), and other groups had no differences (*p* > 0.05) (Table 2).

### 3.4 Serum sex hormone concentrations and knee kinematic parameters

The spearman rank correlations between serum sex hormone concentrations and knee kinematic parameters were shown in Table 3. No significant correlation was observed between serum sex hormone concentrations and knee kinematic parameters in all four groups (*p* > 0.05).

**TABLE 3:** Correlations between sex hormone serum concentrations ranks and knee kinematic parameters ranks in four groups during the menstrual cycle.

	Group 1		Group 2		Group 3		Group 4	
Correlates	r	*p*	r	*p*	r	*p*	r	*p*
Estrogen								
Max flexion	−0.346	0.057	−0.322	0.077	−0.187	0.314	−0.230	0.213
Max extension	0.080	0.668	−0.107	0.565	0.042	0.821	−0.090	0.629
Max varus	−0.170	0.361	−0.261	0.156	−0.136	0.465	−0.263	0.152
Max valgus	0.050	0.788	−0.072	0.702	0.023	0.904	−0.129	0.488
Progesterone								
Max flexion	−0.251	0.174	−0.192	0.301	−0.207	0.264	−0.223	0.227
Max extension	0.021	0.912	0.001	0.994	−0.045	0.808	−0.131	0.484
Max varus	−0.211	0.255	−0.192	0.301	−0.196	0.291	−0.352	0.052
Max valgus	−0.028	0.883	0.053	0.776	−0.068	0.716	−0.151	0.418

^a^ Significant difference between groups (*p* < 0.05).

## 4 Discussion

The study had several findings: 1) The methodology of the menstrual cycle was uncertain and may lead to an insufficient sample size. 2) The biomechanical risk of non-contact ACL injury was lower in the mid-luteal phase when female college soccer athletes performed 90° cutting 3) There was no correlation between serum sex hormone concentrations and knee kinematics parameters.

In the fields of sports medicine and sports science, corresponding conclusions are often contradictory. The main reason is due to the inclusion of anovulatory and LPD (Balachandar et al., 2017; Janse et al., 2019). At present, the proportion of anovulatory and LPD in athletes still lacks large-scale epidemiological surveys. Researchers have speculated that it may be related to lower age, body fat, or longer menstrual cycle (Schaumberg et al., 2017). In physically active females, the incidence of anovulatory and LPD may be as high as 30%. In heavily exercising females, the incidence may exceed 50% (De Souza et al., 2010). Therefore, conducting such a study may result in over half of the subjects being excluded and ultimately facing the problem of insufficient sample size (Janse et al., 2019). The study also confirmed that nearly half of the subjects in female college soccer athletes were excluded.

For the quantitative test of sex hormones, serum indicators are still used as the gold standard. For subjects, it is an invasive test (Allen et al., 2016; Elliott-Sale et al., 2021). In correlation studies related to serum sex hormone concentrations, once the study begins, it means that the test should start from the menstrual phase. Anovulatory is only recognized in the late-follicular phase, while LDP is not recognized until the mid-luteal phase. Therefore, based on reducing the frequency of blood collection and experimental costs, we recommend conducting research in two menstrual cycles.

ACL is a major stabilizing intracapsular ligament in the knee. ACL originates from the medial condyle of the femur and ends in the intercondylar humerus, merging with the anterior horn of the lateral meniscus. Through these attachment points, ACL not only passively limits the advancement of the tibia relative to the femur, but also maintains axial and transverse rotation of the knee. Biomechanical studies have suggested that valgus is one of the important indicators for measuring the risk of non-contact ACL injury (Boden et al., 2010). The greater valgus, the greater load of the ACL. These biomechanical factors may place athletes at a higher risk of non-contact ACL injury (Alentorn-Geli et al., 2009; Quatman and Hewett, 2009; Montalvo et al., 2019). Therefore, we speculated that the biomechanical risk of non-contact ACL injury was lower in the mid-luteal phase when subjects performed 90° cutting.

Cutting and other actions involving changing direction are high-risk actions for non-contact ACL injury and are also hot topics in biomechanical studies. Several studies have suggested that biomechanical indicators exhibited by greater angle cutting actions often indicate a higher risk of non-contact ACL injury (Dos'Santos et al., 2018). In the previous two studies, the angle was 45°, and knee kinematics parameters were not different in different phases (Park et al., 2009a; Park et al., 2009b). Therefore, in the study of cutting actions, we recommend actions with an angle (>45°), which may help identify positive differences. The study found that selecting 90° cutting actions showed a positive difference in knee valgus indicators.

The phase of the menstrual cycle is established on the occurrence of menstruation, follicular maturation, ovulation, and corpus luteum formation. In different phases, estrogen and progesterone show different multiple hormonal milieus (Carmichael et al., 2021). At the beginning of menstruation, estrogen remains at a low level and reaches the first peak in the late-follicular phase, and then drops sharply in the ovulation phase. Estrogen reaches the second peak in the mid-luteal phase and then drops to a lower level in the premenstrual phase. At the beginning of menstruation, progesterone remains at a low level and reaches a peak in the mid-luteal phase, and then decreases to a low level (Meignié et al., 2021).

At present, sex hormone receptors have been found in tissues such as muscles and ligaments (Sung and Kim, 2018). The serum sex hormone concentrations may affect the structure and metabolism of muscles and ligaments, which in turn may affect knee relaxation and neuromuscular control (Nédélec et al., 2021). The study found that there was no correlation between serum sex hormone concentrations and knee kinematics parameters in each phase of the menstrual cycle. The reasonable explanation for the result may be that knee kinematics parameters cannot be simply explained by the serum concentrations of estrogen and progesterone and may be related to other factors (such as neuromuscular control, etc.) (Dargo et al., 2017; Dos'Santos et al., 2018).

The study has some limitations: 1) The subjects’ sports are all female college soccer athletes and cannot represent female athletes in all sports. 2) Due to the selection of cutting angle and the limitations of the experimental equipment, we cannot obtain the transverse plane parameters. 3) The motion analysis system has measurement errors that may lead to false positive results in the frontal plane.

In summary, we used the three-step method to conduct the study, once again validating the risk of sample size uncertainty and the challenge of study in the field. Therefore, we suggested conducting a study during two menstrual cycles. Female college soccer athletes had a lower biomechanical risk of non-contact ACL injury in the mid-luteal phase when performing a larger angle cutting. The evidence helped to increase the understanding of the effect of the menstrual cycle on the biomechanical risk of non-contact ACL injury and to develop corresponding intervention plans. In the in-depth study of the correlation between serum sex hormones concentrations and knee kinematics parameters, no correlation was found between them. Therefore, future study needs to incorporate factors such as neuromuscular control to explore.

## Data Availability

The original contributions presented in the study are included in the article/Supplementary Material, further inquiries can be directed to the corresponding author.
